# Optimism and risk of incident hypertension: a target for primordial prevention

**DOI:** 10.1017/S2045796020000621

**Published:** 2020-08-14

**Authors:** Laura D. Kubzansky, Julia K. Boehm, Andrew R. Allen, Loryana L. Vie, Tiffany E. Ho, Claudia Trudel-Fitzgerald, Hayami K. Koga, Lawrence M. Scheier, Martin E. P. Seligman

**Affiliations:** 1 Department of Social and Behavioral Sciences, Harvard T.H. Chan School of Public Health, Boston, MA, USA; 2 Lee Kum Sheung Center for Health and Happiness, Harvard T.H. Chan School of Public Health, Boston, MA, USA; 3 Department of Psychology, Chapman University, Orange, CA, USA; 4 Department of Psychology, University of Pennsylvania, Philadelphia, PA, USA; 5 Northrop Grumman Technology Services, Seaside, CA, USA; 6 LARS Research Institute, Inc., Scottsdale, AZ, USA

**Keywords:** Attitudes, epidemiology, health outcomes, mental health, prospective study

## Abstract

**Aims:**

Optimism is associated with reduced cardiovascular disease risk; however, few prospective studies have considered optimism in relation to hypertension risk specifically. We investigated whether optimism was associated with a lower risk of developing hypertension in U.S. service members, who are more likely to develop high blood pressure early in life. We also evaluated race/ethnicity, sex and age as potential effect modifiers of these associations.

**Methods:**

Participants were 103 486 hypertension-free U.S. Army active-duty soldiers (mean age 28.96 years, 61.76% White, 20.04% Black, 11.01% Hispanic, 4.09% Asian, and 3.10% others). We assessed optimism, sociodemographic characteristics, health conditions, health behaviours and depression status at baseline (2009–2010) via self-report and administrative records, and ascertained incident hypertension over follow-up (2010–2014) from electronic health records and health assessments. We used Cox proportional hazards regression models to estimate hazard ratios (HRs) and 95% confidence intervals (CIs), and adjusted models for a broad range of relevant covariates.

**Results:**

Over a mean follow-up of 3.51 years, 15 052 incident hypertension cases occurred. The highest *v*. lowest optimism levels were associated with a 22% reduced risk of developing hypertension, after adjusting for all covariates including baseline blood pressure (HR = 0.78; 95% CI = 0.74–0.83). The difference in hypertension risk between the highest *v*. lowest optimism was also maintained when we excluded soldiers with hypertension in the first two years of follow-up and, separately, when we excluded soldiers with prehypertension at baseline. A dose–response relationship was evident with higher optimism associated with a lower relative risk (*p* < 0.001). Higher optimism was consistently associated with a lower risk of developing hypertension across sex, age and most race/ethnicity categories.

**Conclusions:**

In a diverse cohort of initially healthy male and female service members particularly vulnerable to developing hypertension, higher optimism levels were associated with reduced hypertension risk independently of sociodemographic and health factors, a particularly notable finding given the young and healthy population. Results suggest optimism is a health asset and a potential target for public health interventions.

## Introduction

Elevated blood pressure is an established risk factor for developing cardiovascular disease (CVD), the leading cause of morbidity and mortality in the U.S. (Benjamin *et al*., 2017). However, preventing high blood pressure remains a major public health challenge (Chobanian *et al*., 2003). Blood pressure tends to increase with age (Chobanian *et al*., 2003) and current trends show a rising prevalence of hypertension in younger adults (Yano *et al*., 2015). Higher blood pressure in early adulthood persists into later adulthood and inflates CVD risk beyond the effects of hypertension in middle adulthood (Gray *et al*., 2011). Thus, if age-related increases in blood pressure could be prevented or lessened, the burden of CVD might be substantially reduced (Rieder, 2007; Institute of Medicine, 2010). Certain populations have also been identified as having elevated risk of developing hypertension relatively early in life, including those exposed to major occupational stressors, like service members and veterans (Yu *et al*., 2003; Shrestha *et al*., 2019). Besides occupational status, other sociodemographic correlates of high blood pressure have been identified. Higher rates of hypertension occur in younger men *v*. women and early-onset hypertension is more common in Blacks relative to Whites, although less is known about premature risk in other racial groups (e.g. Hispanic and Asian) (Benjamin *et al*., 2017). To date, strategies to reduce hypertension risk have had limited success, with more attention focused on treatment than prevention (Rieder, 2007; Institute of Medicine, 2010). Consequently, recent work has sought to identify modifiable protective factors, particularly for younger, healthy individuals at risk of early-onset hypertension.

Preliminary research has suggested psychological health plays a role in hypertension onset, particularly in the military. For example, one study found a three-fold increase in hypertension risk among veterans with post-traumatic stress disorder (PTSD) or other psychiatric diagnoses; strikingly, these veterans were, on average, 31 years of age (Cohen *et al*., 2009). A national cohort study of 1 547 182 Swedish military male conscripts found those with low *v*. high resilience to stressors at age 18 had over 40% excess hypertension risk over 26 years of follow-up (Crump *et al*., 2016). This work suggests some psychological factors may reduce risk. One such factor is dispositional optimism, the tendency to believe good events are likely and bad events are unlikely (Carver *et al*., 2010). Shaped by social experiences (Boehm *et al*., 2015), optimism tends to be stable starting in young adulthood. However, it is also modifiable via active interventions (Meevissen *et al*., 2011) and has consistently demonstrated protective effects against incident coronary heart disease and stroke (Boehm and Kubzansky, 2012). Most studies of optimism and hypertension have been cross-sectional and conducted with older adults (Trudel-Fitzgerald *et al*., 2015). The only published prospective study found no relationship across 12 years in a sample of mid-life professional adults, although optimism was assessed with only a single item (Trudel-Fitzgerald *et al*., 2014).

Examining optimism's association with incident hypertension risk may provide insight into novel targets for the prevention of a highly prevalent condition. In this study, we hypothesised higher optimism would be associated with reduced risk of developing hypertension in a young and diverse sample of 103 486 U.S. Army active-duty soldiers, a population at high risk of early-onset hypertension (Stewart *et al*., 2015). We used a multi-item measure of dispositional optimism and evaluated associations among initially healthy participants. Following previous research, we included multiple potential confounders (e.g. family history of CVD) and variables that might lie on the pathway linking optimism to hypertension risk (e.g. cigarette smoking) (Cohen *et al*., 2009; Trudel-Fitzgerald *et al*., 2015). We further adjusted for possible confounding by depression, which has been linked with both lower optimism and increased hypertension risk (Trudel-Fitzgerald *et al*., 2015). In secondary analyses, we assessed whether the primary association would hold after accounting for baseline blood pressure and, separately, whether the association differed depending on race/ethnicity, sex or age.

## Methods

### Participants

**Fig. 1.**
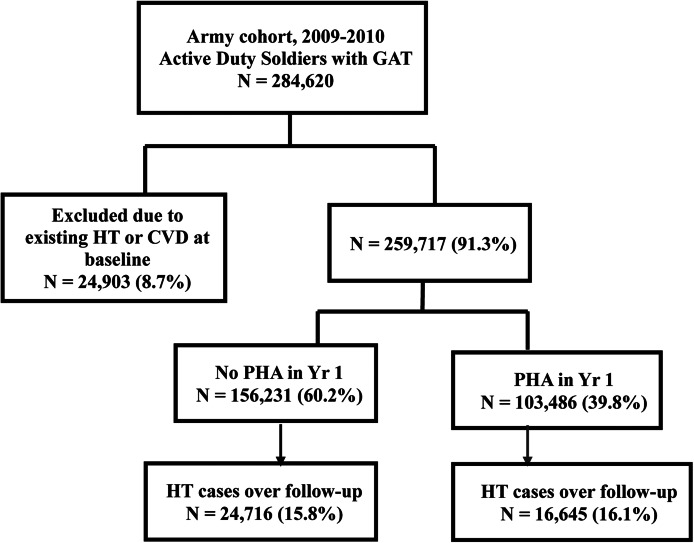
Study population flow chart. Diagram showing how we derived the analytic sample. Soldiers missing a PHA may have completed their physical sometime leading up to or shortly after the specified 12-month baseline window. The PHA includes measures of family history of CVD, diabetes status, BMI, smoking, alcohol misuse, SBP and DBP. GAT: global assessment tool; HT: hypertension; CVD: cardiovascular disease.

Data are from a large cohort of active-duty U.S. Army soldiers followed for up to five years. Every year, soldiers complete the global assessment tool (GAT), a self-report questionnaire assessing psychosocial functioning (Peterson *et al*., 2011). During the study baseline 284 620 Army active-duty soldiers, age 17–65 years, completed the GAT (May 2009–April 2010) and indicated their responses could be used for research purposes. In this population of soldiers with the majority younger than age 30, approximately 8.7% had hypertension at study baseline, which is comparable to estimates of hypertension rates in the civilian population. For example, the National Health and Nutrition Examination Survey reported the prevalence of hypertension in men and women age 20–44 years was 10.5% (AHA Centers for Health Metrics and Evaluation, 13 November 2017). After excluding soldiers with hypertension, suspected hypertension derived from related conditions or CVD at baseline (*n* = 24 903) or without relevant health information at baseline (*n* = 156 231), we retained 103 486 soldiers for analysis (Fig. 1). Comparing soldiers included *v*. excluded because of missing health information [i.e. a missing periodic health assessment (PHA)] at baseline, few differences were evident (see online Supplementary Table S1). Excluded soldiers had more deployments and days deployed, perhaps explaining why they were less likely to have a baseline PHA.

The current study is part of a military–civilian collaboration between the Army and the University of Pennsylvania and was approved by the University of Pennsylvania Institutional Review Board and a DOD Human Research Protection Official.

### Measures

#### Optimism

Optimism was measured in the GAT using a modified version of the revised life orientation test (Scheier *et al*., 1994). Internal consistency was acceptable (*α* = 0.74). After reverse scoring negatively-worded items, we obtained the mean of the item responses and standardised the overall scores (higher scores indicate higher optimism). To assess discontinuous or threshold effects, we also created quintiles of optimism scores based on the distribution of unstandardised scores in the analytic sample (range = 1–5): low (score < 3), low–moderate (score = 3), moderate (3 < score < 4), moderate–high (4 ⩽ score ⩽ 4.5) and high (score > 4.5). Prior work has demonstrated that optimism tends to be stable over time (Scheier and Carver, 2018).

#### Hypertension

Relevant diagnosis and medication data were obtained from electronic health records collected in the Military Health System Data Repository (MDR). The MDR records all outpatient and inpatient medical encounters and medications dispensed to soldiers and reimbursed by TRICARE, the military health insurer (Vie *et al*., 2015). Hypertensive cases through the follow-up period (through 30 April 2014) were defined according to whether a soldier received a hypertension diagnosis using the International Classification of Diseases, Ninth-Revision, Clinical Modification (ICD-9-CM; code 401) or a prescription for antihypertensive medication.

Additionally, measured blood pressure was obtained from the PHA, a health assessment soldiers complete with an Army healthcare provider annually. For some analyses, we considered measured blood pressure as an additional criterion for identifying hypertension cases. Those with blood pressure readings over clinical cut-points of both 140 mmHg [systolic blood pressure (SBP)] and 90 mmHg [diastolic blood pressure (DBP)] were also considered hypertensive. Measured blood pressure was also used to identify individuals with baseline prehypertension (⩾120 mmHg SBP or ⩾80 mmHg DBP).

#### Covariates

Covariates were assessed at baseline unless otherwise indicated. Demographic characteristics obtained from the GAT included sex (men and women), age (years), and rank (officer and enlisted). Other characteristics obtained from electronic administrative records housed at the Defense Manpower Data Center included self-identified race/ethnicity (White, Black, Hispanic, Asian and others), educational attainment (<high school diploma, high school diploma, some college or associate of Arts degree, ⩾college degree) and marital status (married/not married). We used the number of deployments and the total number of days deployed prior to each soldier's baseline GAT (beginning in September 2001), obtained from the Defense Manpower Data Center, to derive a measure of the average length of deployment prior to the baseline GAT. Family history of CVD and participants’ baseline diabetes status were each self-reported with a single item on the PHA. Baseline depression status was assessed according to the presence or absence of a depression diagnosis in the MDR (ICD-9-CM: 296.2, 296.3, 300.4 or 311).

Potential pathway variables included healthy behaviours and related conditions. On the PHA, current cigarette smoking status was assessed using a single self-report item (yes/no) and alcohol misuse was measured with the validated three-item alcohol use disorders identification test (AUDIT-C), which queried alcohol frequency, quantity consumed and binge drinking (Bush *et al*., 1998). Following prior work, AUDIT-C scores were dichotomised using screening thresholds for alcohol misuse (⩾4 in men; ⩾3 in women) (Bradley *et al*., 2007). Body mass index (BMI) in kg/m^2^ was derived from height and weight measurements taken by an Army professional close in time to the soldier's baseline PHA. Given stringent muscular strength, endurance and cardiovascular respiratory fitness requirements for active-duty Army soldiers, we did not adjust for physical activity.

### Statistical analyses

We analysed data in the person-event data environment, a secure, cloud-based environment that houses Army workforce, medical and personnel data (Vie *et al*., 2015). Using SAS 9.4, Cox proportional-hazard models estimated the hazard ratios (HRs) and 95% confidence intervals (CIs) for associations between optimism, assessed at the 2009–2010 baseline, and incident hypertension. Soldiers were followed until diagnosis of hypertension, separation from Army service, death or end of follow-up (fixed censoring date of 30 April 2014), whichever occurred first. Multiple imputation procedures were used to handle missing values on BMI (missing: 31.45%), as well as other variables with fewer missing values (⩽1%). Parameter estimates from ten imputed datasets were pooled (Schafer, 1999).

We examined three nested models: (1) an age-adjusted model; (2) a confounder model that was adjusted for age, demographic factors (sex, race/ethnicity, education and marital status) and other potential confounders (officer rank, average length of deployment, diabetes status, family history of CVD and depression status); and (3) a pathway model that included all variables from model 2 along with BMI, cigarette smoking status and alcohol misuse. Separate model sets were run using optimism either as a standardised, continuous variable or as quintiles to assess possible discontinuous effects. An examination of the correlation between the Schoenfeld residuals and functions of time in the primary model supported the assumption of proportional hazards (the highest absolute correlation was 0.05) (Schoenfeld, 1982).

We ran three additional sets of analyses to refine the primary models described above. First, we examined the role of blood pressure in two ways: we added SBP and DBP as additional covariates in models 1–3 and, separately, we included blood pressure levels as a defining criterion for hypertension in the covariate-adjusted model (model 2). Second, to assess if effects were similar across demographic subgroups when adjusting for confounders, we included an interaction term (or terms) for optimism (continuous) with race/ethnicity (White, Black, Hispanic, Asian and others), sex (men and women) or age (continuous). We also assessed potential effect modification by running separate confounder-adjusted models stratified by race/ethnicity, sex, or age (⩽30 and >30 years). Third, to reduce concerns about reverse causality (i.e. subclinical hypertension influencing optimism levels), we ran each model excluding hypertension cases that developed within two years of baseline. Separately, we also ran the three main nested models excluding individuals with prehypertension at baseline.

## Results

At the 2009–2010 baseline, a majority of soldiers (64%) were ⩽30 years of age (mean age 28.96 years, range = 17–65), men (83.24%) and White (61.76%). Mean BMI was 26.58 kg/m^2^ [standard deviation (s.d.) = 3.66], 24% of participants reported a family history of CVD and very few reported diabetes (0.29%). Table 1 shows the distribution of covariates across optimism quintiles. Individuals with high optimism levels were more likely to be older, have at least a college education, be an officer, not be depressed, not be a current smoker and not misuse alcohol. Over a mean follow-up period of 3.51 years (s.d. = 1.23), 15 052 soldiers developed hypertension (14.54%). Notably, soldiers with hypertension at baseline (i.e. those excluded from this study) had nearly identical optimism scores as the hypertension-free soldiers included in this study (3.59 *v*. 3.61).

**Table 1.** Distribution of covariates at baseline (2009–2010) according to the levels of optimism

Characteristic	Q1 low	Q2 low–moderate	Q3 moderate	Q4 moderate–high	Q5 high
Number of soldiers	15 731	20 990	25 804	24 839	16 122
Age, in years: *M* (s.d.)	27.41 (6.73)	27.61 (6.83)	28.67 (7.39)	29.76 (7.83)	31.47 (8.26)
Sex: *n* (%)					
Male	13 351 (84.87)	18 167 (86.55)	21 616 (83.77)	20 110 (80.96)	12 893 (79.97)
Female	2380 (15.13)	2823 (13.45)	4188 (16.23)	4729 (19.04)	3229 (20.03)
Race/ethnicity: *n* (%)					
White	11 258 (71.57)	12 974 (61.81)	15 741 (61.00)	14 757 (59.42)	9178 (56.93)
Black	2152 (13.68)	4060 (19.34)	4805 (18.62)	5455 (21.96)	4271 (26.49)
Hispanic	1416 (9.00)	2342 (11.16)	3132 (12.14)	2804 (11.29)	1697 (10.53)
Asian	503 (3.20)	976 (4.65)	1267 (4.91)	1029 (4.14)	456 (2.83)
Others	402 (2.56)	637 (3.03)	859 (3.33)	792 (3.19)	520 (3.23)
Education: *n* (%)					
<High school diploma	177 (1.14)	218 (1.05)	248 (0.97)	205 (0.83)	127 (0.79)
High school diploma	13 135 (84.34)	17 191 (82.63)	19 445 (76.06)	16 932 (68.76)	9872 (61.73)
Some college	592 (3.80)	778 (3.74)	1128 (4.41)	1231 (5.00)	840 (5.25)
College or beyond	1669 (10.72)	2619 (12.59)	4745 (18.56)	6258 (25.41)	5152 (32.22)
Marital status: *n* (%)					
Married	8767 (55.73)	11 636 (55.44)	15 154 (58.73)	15 320 (61.68)	10 665 (66.15)
Unmarried	6964 (44.27)	9354 (44.56)	10 650 (41.27)	9519 (38.32)	5457 (33.85)
Rank: *n* (%)					
Officer	1352 (8.60)	2159 (10.30)	4095 (15.89)	5584 (22.54)	4630 (28.79)
Enlisted	14 362 (91.40)	18 804 (89.70)	21 675 (84.11)	19 194 (77.46)	11 452 (71.21)
Pre-baseline:					
Deployments: *M* (s.d.)	0.97 (1.06)	0.97 (1.06)	1.00 (1.11)	1.00 (1.12)	1.03 (1.15)
Days deployed: *M* (s.d.)	294.33(321.86)	296.44(324.28)	295.88(326.48)	291.44(324.01)	290.74(318.65)
Diabetes: *n* (%)	49 (0.31)	65 (0.31)	65 (0.25)	69 (0.28)	49 (0.30)
Family history of CVD: *n* (%)	3926 (24.96)	4067 (19.38)	6283 (24.35)	6487 (26.12)	4562 (28.30)
Depression: *n* (%)	2039 (12.96)	1344 (6.40)	1265 (4.90)	872 (3.51)	422 (2.62)
BMI: *M* (s.d.)	26.75 (3.89)	26.61 (3.70)	26.53 (3.66)	26.51 (3.59)	26.57 (3.51)
Current smoker: *n* (%)	6173 (39.24)	6900 (32.87)	7670 (29.72)	6091 (24.52)	3287 (20.39)
Alcohol misuse: *n* (%)	5514 (35.05)	6760 (32.21)	7959 (30.84)	6939 (27.94)	3696 (22.93)
SBP: *M* (s.d.)	122.56 (11.53)	122.67 (11.37)	122.45 (11.45)	122.36 (11.51)	122.46 (11.64)
DBP: *M* (s.d.)	73.70 (9.25)	73.70 (9.24)	73.79 (9.23)	73.88 (9.19)	74.14 (9.28)

Values are either mean (s.d.) or *n* (%). *N* = 103 486.

Note: non-imputed data are reported for descriptive purposes. Percentages refer to the column percent of individuals within each optimism category with that characteristic.

BMI, body mass index; BP, blood pressure; CVD, cardiovascular disease; Q, quintile; s.d., standard deviation.

### Optimism and incident hypertension

In the age-adjusted model, we observed a 13% decreased risk of incident hypertension with each 1-s.d. increase in the optimism score (HR = 0.87; 95% CI = 0.85–0.88). This estimate was slightly attenuated but remained statistically significant after adjusting for demographics and health conditions, as well as for healthy behaviours that might lie on the pathway (see Table 2 and online Supplementary S2). It is noteworthy that optimism was associated with reduced risk of hypertension independent of depression diagnosis (also significantly associated with higher hypertension risk; HR = 1.66; 95% CI = 1.56–1.76).

**Table 2.** HRs (95% CIs) for the association between optimism and incident hypertension, 2009–2014

Variable	Number of cases	Model 1^a^ HR (95% CI)	Model 2^b^ HR (95% CI)	Model 3^c^ HR (95% CI)
Optimism (continuous)	15 052	0.87 (0.85–0.88)***	0.91 (0.89–0.92)***	0.92 (0.91–0.94)***
Optimism (quintiles)				
Q1 (low)	2492	1.00	1.00	1.00
Q2 (low–moderate)	3127	0.89 (0.84–0.94)***	0.94 (0.89–0.99)*	0.95 (0.90–1.00)
Q3 (moderate)	3680	0.78 (0.74–0.82)***	0.85 (0.80–0.89)***	0.87 (0.82–0.91)***
Q4 (moderate–high)	3479	0.71 (0.68–0.75)***	0.80 (0.76–0.85)***	0.83 (0.79–0.87)***
Q5 (high)	2274	0.66 (0.62–0.70)***	0.76 (0.72–0.81)***	0.79 (0.75–0.84)***

*N* = 103 486.

HR, hazard ratio; CI, confidence interval, Q: quintile.

a Adjusted for age.

b Adjusted for age, sex, race/ethnicity, education, marital status, rank, average deployment length prior to baseline, diabetes status, family history of CVD and depression.

c Adjusted for model 2 covariates as well as BMI, smoking status and alcohol misuse.

*p < 0.05, **p < 0.01, ***p < 0.001.

**Fig. 2.**
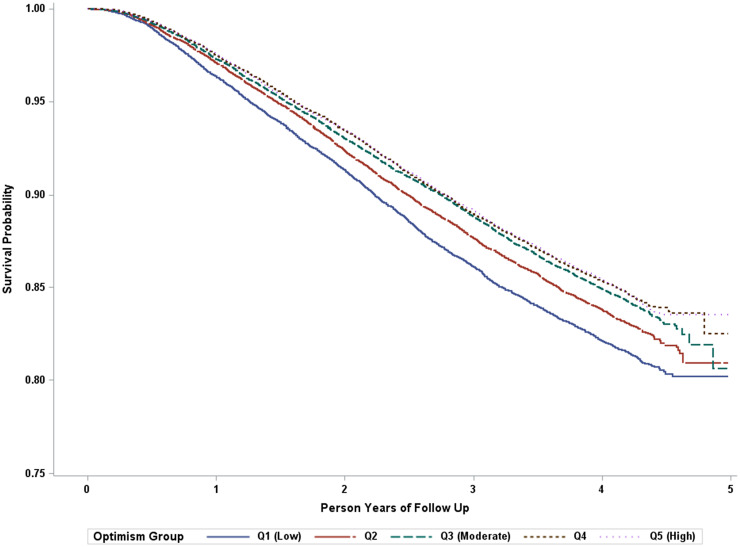
Unadjusted Kaplan–Meier survival curve for optimism and incident hypertension. Estimated survival probability of incident hypertension according to the level of optimism, with individuals with higher optimism at lower risk of developing hypertension.

Additionally, findings with optimism quintiles demonstrated a 34% reduction in subsequent hypertension risk for the highest *v*. lowest optimism quintiles in the age-adjusted model (HR = 0.66; 95% CI = 0.62–0.70). Relative to the lowest optimism quintile, all other optimism quintiles were also significantly associated with a reduced risk of hypertension in the age-adjusted model. After further adjusting for potential confounders, a dose–response relationship remained evident with each increase in optimism quintile associated with a lower relative risk (*p* for trend <0.001). Optimism's association with hypertension risk remained statistically significant but was somewhat attenuated after additionally adjusting for healthy behaviours that might lie on the pathway (see Table 2). Figure 2 illustrates the estimated survival probability of incident hypertension for individuals within each optimism quintile.

### Additional analyses

Adding measured levels of SBP and DBP to our primary models demonstrated higher SBP (HR = 1.02; 95% CI = 1.02–1.02) and DBP (HR = 1.03; 95% CI = 1.02–1.03) were each uniquely associated with increased hypertension risk in the confounder-adjusted model, and findings were virtually identical in fully adjusted models. However, even after including measured SBP and DBP at baseline in the confounder-adjusted model, optimism remained significantly associated with lower hypertension risk (per 1-s.d., HR = 0.91; 95% CI = 0.90–0.93; highest *v*. lowest optimism quintile, HR = 0.76; 95% CI = 0.72–0.81). See Table 3. Additionally, in separate confounder-adjusted models, optimism remained significantly associated with lower hypertension risk when thresholds according to measured SBP and DBP were included as part of the defining criteria for hypertension case status (per 1-s.d. optimism, HR = 0.92; 95% CI = 0.90–0.93; highest *v*. lowest optimism quintile, HR = 0.78; 95% CI = 0.74–0.83).

**Table 3.** Association between optimism and incident hypertension while adjusting for baseline SBP and DBP, 2009–2014

Variable	Number of cases	Model 1^a^ HR (95% CI)	Model 2^b^ HR (95% CI)	Model 3^c^ HR (95% CI)
Optimism (continuous)	15 052	0.88 (0.86–0.89)***	0.91 (0.90–0.93)***	0.92 (0.90–0.93)***
Optimism (quintiles)				
Q1 (low)	2492	1.00	1.00	1.00
Q2 (low–moderate)	3127	0.89 (0.84–0.94)***	0.94 (0.89–0.99)*	0.95 (0.90–1.00)
Q3 (moderate)	3680	0.79 (0.75–0.83)***	0.85 (0.80–0.89)***	0.86 (0.82–0.91)***
Q4 (moderate–high)	3479	0.73 (0.69–0.77)***	0.81 (0.76–0.85)***	0.83 (0.78–0.87)***
Q5 (High)	2274	0.68 (0.64–0.72)***	0.76 (0.72–0.81)***	0.78 (0.74–0.83)***

*N* = 103 486.

HR, hazard ratio; CI, confidence interval, Q: quintile.

a Adjusted for age, SBP and DBP.

b Adjusted for age, SBP, DBP, sex, race/ethnicity, education, marital status, rank, average deployment length prior to baseline, diabetes status, family history of CVD and depression.

c Adjusted for model 2 covariates as well as BMI, smoking status and alcohol misuse.

*p < 0.05, **p < 0.01, ***p < 0.001.

We observed a statistically significant interaction of optimism with age (*p* = 0.01) and with being Black (*p* < 0.001) compared to being White, but not with sex (*p* = 0.58) or with being Asian (*p* = 0.29). In age-stratified analyses, associations of optimism with the risk of developing hypertension were somewhat stronger in soldiers >30 years relative to younger soldiers (Table 4). Although associations of optimism with hypertension risk were largely similar across racial/ethnic groups relative to Whites (Table 4), the HR for optimism was slightly attenuated (but still statistically significant) for Blacks. Optimism was not significantly associated with hypertension risk among Asians; however, there were fewer cases of hypertension documented among Asians during follow-up (*n* = 450), relative to other racial/ethnic groups.

**Table 4.** Association between optimism (per 1 s.d.) and incident hypertension, modelled separately within categories of race/ethnicity, sex and age, 2009–2014

Demographic group	*n* (*n* cases)	HR (95% CI)
Race/ethnicity^a^		
White	63 908 (8704)	0.90 (0.88–0.92)***
Black	20 743 (3806)	0.93 (0.90–0.96)***
Hispanic	11 391 (1539)	0.90 (0.85–0.95)***
Asian	4231 (450)	0.93 (0.84–1.04)
Others	3210 (553)	0.89 (0.81–0.97)**
Sex^b^		
Male	86 137 (12 486)	0.91 (0.90–0.93)***
Female	17 349 (2566)	0.90 (0.86–0.94)***
Age^c^		
30 years and younger	66 138 (7698)	0.93 (0.91–0.95)***
Over 30 years	37 345 (7354)	0.88 (0.86–0.91)***

HR, hazard ratio; CI, confidence interval, s.d., standard deviation.

a Adjusted for demographics (age, sex, education and marital status), rank, average deployment length prior to baseline, diabetes status, family history of CVD and depression.

b Adjusted for demographics (age, race/ethnicity, education and marital status), rank, average deployment length prior to baseline, diabetes status, family history of CVD and depression.

c Adjusted for demographics (age, sex, race/ethnicity, education and marital status), rank, average deployment length prior to baseline, diabetes status, family history of CVD and depression.

*p < 0.05, **p < 0.01, ***p < 0.001.

After excluding hypertension cases that developed within the first two years of baseline, optimism remained associated with an increased risk of developing hypertension. Specifically, after adjusting for potential confounders (i.e. model 2), estimates were somewhat attenuated but still statistically significant (per 1-s.d., HR = 0.93; 95% CI = 0.91–0.95; highest *v*. lowest optimism quintile, HR = 0.81; 95% CI = 0.75–0.88). Similarly, optimism remained significantly associated with hypertension risk after excluding individuals with prehypertension at baseline (model 2, per 1-s.d. of optimism, HR = 0.88; 95% CI = 0.85–0.91; highest *v*. lowest optimism quintile, HR = 0.71; 95% CI = 0.63–0.80).

## Discussion

This study provides consistent evidence that optimism is prospectively associated with reduced risk of hypertension over a four-year period in a large sample of initially healthy Army soldiers. Particularly noteworthy is that higher optimism levels were associated with reduced risk across a diverse set of individuals, including young men and women, as well as Blacks, Whites, and Hispanics. Effects were somewhat stronger in individuals over age 30, but still evident among younger soldiers. Moreover, associations were independent of conventional cardiovascular risk factors (including measured baseline blood pressure levels), length of deployment, Army rank and depression. Given depression was also significantly associated with increased hypertension risk; our findings may suggest optimism leads to health benefits beyond simply marking the absence of depression or other mental health disorders. Including health behaviours attenuated associations somewhat; however, estimates remained statistically significant, suggesting the possibility of other mechanisms at play. These findings are consistent with other research demonstrating independent associations of optimism with other cardiovascular outcomes including coronary heart disease (Boehm and Kubzansky, 2012; Carver and Scheier, 2014).

The rate of incident hypertension in the current sample (14.5%) is higher than has been noted in civilian populations of comparable age. For example, in participants age 18–30 entering the coronary artery risk in young adults study, the ten-year incidence of hypertension ranged from 3.2% in White women to 7.8% in White men and 16.4% in Black men (Dyer *et al*., 1999). However, other research suggests soldiers exposed to high levels of combat stressors and related distress develop hypertension at higher rates. For example, one study (*n* =  303 223; mean age 31 years) reported 15.6% prevalence of hypertension among U.S. veterans with a mental health diagnosis excluding PTSD and 30.8% prevalence among those with PTSD with or without another mental health diagnosis (Cohen *et al*., 2009). In a cross-sectional study also drawing on data from active-duty U.S. Army soldiers but weighted to the 2012 U.S. Army active-duty population, 5% of soldiers were estimated to have hypertension and 64.7% were estimated to be pre-hypertensive at the time of assessment in 2012 (Shrestha *et al*., 2019). Thus, findings from this sample do support the notion that individuals in the military are at higher risk of developing hypertension relatively early in life.

One key pathway linking optimism with reduced hypertension risk may operate by increasing healthy behaviours. Numerous studies indicate optimists are more likely to have healthier diets, exercise more and obtain adequate sleep (Kelloniemi *et al*., 2005; Steptoe *et al*., 2006; Trudel-Fitzgerald *et al*., 2019), while less likely to engage in behaviours that jeopardise health, like excess alcohol consumption or smoking (Carvajal *et al*., 2000; Steptoe *et al*., 2006; Trudel-Fitzgerald *et al*., 2019). A recent meta-analysis found more *v*. less optimistic individuals tend to engage in healthier behaviours that protect against CVD, although effect sizes were modest (Boehm *et al*., 2018). In line with these findings, we found healthy behaviours partially but not fully attenuated associations in our study. Optimism may also increase the likelihood of healthier biological function by buffering the cardiotoxic effects of stressors and psychological distress or improving endothelial function (Kubzansky *et al*., 2018).

Because many of the conventional risk factors for hypertension and CVD – including diet, exercise, and obesity – are difficult to change (Fjeldsoe *et al*., 2011), identifying an upstream determinant of behaviour-related factors may offer critical insights for interventions to reduce risk. Optimism has been described as a unique factor that directly contributes to the transformation of goals into behaviours (Scheier and Carver, 1987). It is partially heritable (Plomin *et al*., 1992) but can also be learned (Murphy *et al*., 2015; Peters *et al*., 2010) and changed over extended time periods, especially in adulthood (Mroczek, 2014). Moreover, optimism can be modified intentionally with relatively low-burden strategies, including expressing gratitude or meditating, as well as by more resource-intensive strategies like engaging in cognitive-behavioural or well-being prevention and therapy programmes (Peters *et al*., 2010; Ruini and Fava, 2012). A critical next step is to evaluate if interventions to improve optimism translate into health benefits.

The present study has some limitations including a relatively short follow-up period. Because our sample comprised a high proportion of men with high school level education and good initial physical condition, this study's internal validity is enhanced, but findings may not be generalisable. Due to the stability of optimism scores across years (Scheier and Carver, 2018) and the brief follow-up period, we could not assess whether a change in optimism was associated with altered risk of subsequently developing hypertension. Given that optimism remained a significant predictor of hypertension risk even after adjusting for measured blood pressure at baseline, removing hypertension cases within two years of baseline, and, separately, removing prehypertension cases at baseline, it is unlikely hypertension played a causal role in observed optimism levels. Current findings are based on observational data and cannot rule out the possibility of unmeasured confounding. However, with longer follow-up, future studies may assess whether changes in optimism affect hypertension risk, providing stronger evidence for a causal relationship. Additionally, because this study relied on measured blood pressure obtained from medical records, assessed as part of a clinical examination, this measure may not be directly comparable to one obtained solely for research purposes. However, empirical tests of the measure were reassuring, as we found the association between optimism and HT held when blood pressure was added as an indicator of HT, and blood pressure measures were associated with HT risk, as obtained from the MDR. Methodological strengths include an examination of a large, diverse sample of younger adults serving in the Army uniquely at risk for hypertension. This is one of the first studies to examine associations not only in Whites and Blacks but also in Hispanics and Asians. Moreover, the study was prospective and assessed numerous potential confounding and pathway variables.

## Conclusion

Given that hypertension developed in younger adulthood may be particularly pernicious and that individuals involved in stressful jobs may be at elevated risk for early-onset hypertension (Cuevas *et al*., 2017), identifying protective factors that reduce risk in these populations is urgent. Rates of incident hypertension in this population of U.S. soldiers were striking. Nonetheless, we found higher optimism was associated with reduced incident hypertension risk, over and above a range of potential confounders and pathway-related variables. Protective associations of optimism were evident even within these young and initially healthy individuals who were likely exposed to trauma and stressors over follow-up. While the effect size was modest, the consistency of the associations across multiple racial/ethnic, age and sex groups suggests findings are robust. Prior work indicates that every 10 mmHg SBP reduction leads to approximately 20% reduction in CVD risk, 27% reduction in stroke risk and 17% reduction in coronary heart disease risk (Ettehad *et al*., 2016). We found for every s.d. of optimism, hypertension risk was reduced by 8–13% depending on the covariates included. Identifying individuals with lower optimism levels in early adulthood may serve not only as a sign that action is needed to improve their psychological health but also as the ‘canary in the coal mine,’ providing an early alarm that lifelong cardiovascular health may be at risk. These findings, together with other work demonstrating optimism is a modifiable health asset, suggest optimism may be a valuable target for public health intervention.

## Supplementary material

For supplementary material accompanying this paper visit http://dx.doi.org/10.1017/S2045796020000621. click here to view supplementary material

## Availability of data and materials

Under US Army policy, the data will not be made available to other researchers for purposes of reproducing the results.
