# Supplementary material for: Optimism and risk of incident hypertension: a target for primordial prevention
Source: Epidemiol Psychiatr Sci. 2020 Aug 14;29:e157. doi: 10.1017/S2045796020000621 (PMC7443774; doi:10.1017/S2045796020000621)
Supplement: Supplementary file 1 [file S2045796020000621sup001.docx]

**SUPPLEMENT**

Table S1. Distribution of covariates at baseline (2009-2010), according to inclusion status. Values are either mean (SD) or n (%). N=259,717

| Characteristic | Excluded  (Missing PHA) | Included |
| --- | --- | --- |
| Number of soldiers | 156,231 | 103,486 |
| Optimism: *M (SD)* | 3.59 (0.87) | 3.61 (0.87) |
| Age, in years: *M (SD)* | 28.46 (7.35) | 28.96 (7.57) |
| Sex: *n (%)* |  |  |
| Male | 135,070 (86.46) | 86,137 (83.24) |
| Female | 21,161 (13.54) | 17,349 (16.76) |
| Race/Ethnicity: *n (%)* |  |  |
| White | 99,367 (63.60) | 63,908 (61.76) |
| Black | 29,264 (18.73) | 20,743 (20.04) |
| Hispanic | 17,374 (11.12) | 11,391 (11.01) |
| Asian | 5,713 (3.66) | 4,231 (4.09) |
| Other | 4,509 (2.89) | 3,210 (3.10) |
| Education: *n (%)* |  |  |
| <High school diploma | 1,353 (0.87) | 975 (0.95) |
| High school diploma | 120,543 (77.88) | 76,575 (74.66) |
| Some college | 6,435 (4.16) | 4,569 (4.45) |
| College or beyond | 26,444 (17.09) | 20,443 (19.93) |
| Marital status: *n (%)* |  |  |
| Married | 89,898 (57.54) | 61,542 (59.47) |
| Other | 66,333 (42.46) | 41,944 (40.53) |
| Rank: *n (%)* |  |  |
| Officer | 24,705 (15.84) | 17,820 (17.25) |
| Enlisted | 131,249 (84.16) | 85,487 (82.75) |
| Pre-baseline: |  |  |
| Deployments: *M (SD)* | 1.27 (1.17) | 0.99 (1.10) |
| Days deployed: *M (SD)* | 387.57 (351.41) | 292.43 (322.90) |
| Diabetes: *n (%)* | Missing | 297 (0.29) |
| Family history of CVD: *n (%)* | Missing | 25,326 (24.47) |
| Depression: *n (%)* | 7,757 (4.97) | 5,942 (5.74) |
| BMI: *M (SD)* | 26.62 (3.65) | 26.58 (3.66) |
| Current smoker: *n (%)* | Missing | 30,121 (29.11) |
| Alcohol misuse: *n (%)* | Missing | 30,868 (29.83) |
| BP, Systolic: *M (SD)* | Missing | 122.49 (11.49) |
| BP, Diastolic: *M (SD)* | Missing | 73.83 (9.23) |

*Note*. Non-imputed data are reported for descriptive purposes. Percentages refer to the column percent of individuals within each status with that characteristic. CVD: cardiovascular disease; SD: standard deviation
